# Knowledge and Practice on Breast Self-Examination and Associated Factors among Summer Class Social Science Undergraduate Female Students in the University of Gondar, Northwest Ethiopia

**DOI:** 10.1155/2021/8162047

**Published:** 2021-04-09

**Authors:** Muhabaw Shumye Mihret, Temesgen Worku Gudayu, Abera Shiferaw Abebe, Emebet Gebru Tarekegn, Solomon Ketemaw Abebe, Mosina Aminu Abduselam, Tejitu Dereje Shiferaw, Genet Worku Kebede

**Affiliations:** ^1^Department of Clinical Midwifery, School of Midwifery, College of Medicine and Health Sciences, University of Gondar, Gondar, Ethiopia; ^2^Blue Nile College, Gondar, Ethiopia; ^3^University of Gondar Comprehensive Specialized Hospital, Gondar, Ethiopia; ^4^Ambagiyorgis Health Center, Amhara Regional Health Bureau, Ethiopia; ^5^Mussiebamb Health Center, Amhara Regional Health Bureau, Ethiopia

## Abstract

**Background:**

Breast cancer is a global health concern and a leading cause of morbidity and mortality among women. Early detection of breast cancer contributes to timely linkage to care and reduction of complications associated with breast cancer. In this context, breast self-examination is helpful to detect breast abnormalities particularly in settings with poor access to healthcare for clinical breast examination and mammography. Thus, all women in such settings are highly encouraged to perform breast self-examination regularly, and shreds of evidences are compulsory in this perspective. In the study setting, there was a scarcity of evidence on breast self-examination. Therefore, this study is aimed at assessing knowledge and practice on breast self-examination and its associated factors among summer class female students of social science at Maraki Campus, University of Gondar, Ethiopia.

**Methods:**

An institution-based cross-sectional study was conducted from July 01 to September 15/2018. A total of 398 female summer students were included in the study. A simple random sampling technique was utilized to select the study participants, and interviewer-administered structured questionnaires were employed to collect the data. The data were then entered into Epi info version 7.0, and analysis was done by SPSS version 20.0. A bivariable and multivariable logistic regression model was fitted, and the level of significance was declared based on adjusted odds ratio with its 95% CI and a *p* value ≤ 0.05.

**Result:**

The proportion of students having a good knowledge and practice of breast self-examination was found to be 27.6% (95% CI: 22.9, 32) and 17.4% (95% CI: 13.8, 21.6), respectively. In the multivariable logistic regression analysis, urban residency (AOR = 2.50; 95% CI: 1.27, 4.94) and discussion with someone on breast self-examination (AOR = 4.57; 95% CI: 2.42, 8.65) were predictors of good knowledge, whereas family history of breast cancer (AOR = 7.14; 95% CI: 1.75, 25), discussion with someone on breast self-examination (AOR = 3.85; 95% CI: 1.82, 8.33), and good knowledge on breast self -examination (AOR = 12.02; 95% CI: 5.97, 24.20) had been significantly associated with breast self-examination practice.

**Conclusion:**

In this study, the proportion of students with good knowledge and practice towards breast self-examination was lower than most of the studies done so far. The predictors of breast self-examination are related to lack of information. Thus, awareness creation on breast self-examination would be helpful in this context.

## 1. Introduction

Breast cancer (BC) is a global health concern and a leading cause of morbidity and mortality among women [1]. It is among the top three leading causes of cancers and top five causes of deaths of cancers among women [2]. Globally, BC recent evidences exhibit that about 25% of new cancer cases and 15% of cancer deaths among females in globe are due to BC [3]. For instance, in 2018, it is estimated that about 627,000 women died from BC worldwide [4]. Breast cancer incidence rate (BCIR) remains highest in more developed regions, but mortality is relatively much higher in less developed countries [3, 4]. This is mostly due to lack of advanced laboratory investigations for early screening and diagnosing BC in resource limited countries [5].

Early detection of BC plays a key role for better outcomes, and BC is preventable and treatable if it is detected and treated early [4]. In this aspect, there are two early detection strategies for BC. These are early diagnosis and screening. Screening consists of testing women to identify cancers before any symptom appears and can be undertaken through mammography, clinical breast exam (CBE), and breast self-exam (BSE) [3, 4]. Although clinical breast examination and mammography are ideal for BC diagnosis, access to healthcare in most sub-Saharan African (SSA) countries may be a major challenge. Therefore, BSE is the most feasible option in such settings as it is easily workable, at any place and time [2, 4]. Therefore, efforts need to be harmonized to increase awareness and practice of BSE among women. In this regard, gathering reliable and representative data on each segment of the population is an integral part of generating evidence-based strategies to improve women's health thereby to achieve target four of Sustainable Development Goal (SDG) three [6].

A recent scoping review on BSE among women in SSA demonstrates limited published research and revealed varied level of knowledge ranging from 8.7% [5] to 98.9% [7] and practice ranging from 11.7% to 78%. It further suggests that BSE practice remains a challenge in SSA [8]. In Ethiopia, studies regarding BSE largely focused on health care workers and health science students and were conducted in large cities of the country mainly at referral hospitals [9–13]. In this context, a systematic review (SR) and meta-analysis (MA) on BSE practice among female health care workers depicts that the pooled prevalence of BSE is 56.31% ranging from 32.5% to 80.7% and predicted by good knowledge, positive attitude, and family history of BC [14]. A more recent review article on BSE among all women in Ethiopia shows that the pooled BSE practice is 36.72% [15]. The regional distribution BSE ranged from 21.2% in Tigray to 61.5% in Gambela, whereas the pooled BSE practice in Amhara region is reported to be 40.5% [15]. However, as to the authors' deep review, there were limited shreds of evidences regarding BSE among social science and summer students in particular. Therefore, the current study is aimed at assessing knowledge and practice on BSE and associated factors among summer female social science students at Maraki Campus, University of Gondar, Northwest Ethiopia, 2018.

## 2. Methods

### 2.1. Study Design and Setting

An institution-based cross-sectional study was conducted from July 01 to September 15/2018. The study was carried out at Maraki Campus of University of Gondar which is located in Amhara national regional state at about 740 kilometers away from Addis Ababa, the capital city of Ethiopia. The University of Gondar (UoG) is one of the famous higher education institutions in Ethiopia. The University has five campuses, namely, College of Medicine and Health Sciences (CMHS), Tewodros Campus (Campus of Natural Sciences), Maraki Campus (Campus of Social Sciences), Fasil Campus (Campus of Technology), and Teda Campus (Campus of Agriculture). Maraki Campus was selected because it is the only campus of social science students who are less likely to get health-related information through their academic courses as compared to the natural science students.

### 2.2. Study Population

In Maraki Campus, a total of 2,607 female students have been registered for summer program in different schools and colleges in 2018. Summer female students whose name had been registered at the registrar of Maraki Campus during the study period had been included in the study, whereas those female students who had been registered but leaving the campus due to different reasons had been excluded.

### 2.3. Sample Size Determination and Sampling Technique

Sample size was determined for each specific objective by using Open-Epi Version 3 software. Accordingly, the largest sample size was found to be 362 which was calculated with the following assumptions: ratio between female students knowing the three positions of BSE and those students who were not aware of the three positions of BSE—1 : 1, odds ratio—2.25, power—80%, confidence interval—95%, and percent of unexposed group with outcome—12.8% and that of exposed group with outcome—50.3% [16]. After adding 10% nonresponse rate, the final sample size was turned to be 398.

The list of all female summer students had been obtained from the Campus's registrar. After allocating students' list to each college and school proportionally, the desired samples had been selected by using simple random sampling technique.

### 2.4. Study Variables

This study has two outcome variables. These are knowledge and practice of BSE.

The explanatory variables included sociodemographic and general students' characteristics like age, residence, educational status, occupation, marital status, family history of breast cancer, prenatal visit, personal history of breast cancer, personal history of breast complication, history of contraceptive use, access to media, discussion with someone on the importance of BSE, and knowledge on BSE.

### 2.5. Operational Definitions

Knowledge on BSE status was defined as awareness of students regarding BSE as measured by a structured knowledge questionnaire on BSE. Accordingly, it had been dichotomized as good knowledge (a score of ≥40% correct responses) and poor knowledge (a score less than 40% of the correct responses) [17]. Similarly, about ten criteria (checklists) had been employed to assess the respondents' BSE practice. Accordingly, the cut-off point for good BSE practice is ≥40% of correct response, whereas that of poor BSE practice is <40% [17].

### 2.6. Data Quality Management and Collection Methods

Data were collected by trained female health care workers through an interviewer-administered structured questionnaire. The questionnaire was prepared in English language and translated into local (Amharic) language and back to English to check for accuracy and consistency. Pretest was done on 5% of the sample size among female students at the natural science campus prior to the commencement of actual data collection.

### 2.7. Data Analysis

The data had been checked for its completeness and missing information before leaving the participants. Thereafter, data were entered into Epi Info version 7 and then exported to SPSS version 20 for analysis. A bivariable and multivariable regression model was fitted. All explanatory variables with a *p* value of <0.2 in the bivariable logistic regression analysis were candidates for the multivariable logistic regression model. Finally, statistically significant association had been declared based on AOR with 95% CI and its *p* value of ≤0.05.

## 3. Result

### 3.1. Sociodemographic and General Characteristics of the Participants

A total of 384 participants responded to the questionnaire making the response rate of 96.5%. The age of the study participants ranged from 20 to 45 years with the median age of 28 years (IQR = 6). The majority (56.8%) of the respondents were urban residents. More than half (58.1%) of the participants had ever married. Most (78.9%) of the respondents were government employees, and more than half of the respondents (53.4%) were self-sponsored for education. And one-third of the respondent was third year students.

Considerable proportions (5.2%) of the respondents reported that they had a family history of BC, and about 25 (7.0%) of the participants had a history of breast complication. Nearly half (48.2%) of the students had used contraceptive methods, and nearly one-sixth (16.2%) of the respondents had ever discussed with someone on the importance of BSE **(**Table 1).

### 3.2. Knowledge and Practice on BSE

The proportion of students reporting good knowledge on BSE was obtained to be 27.6% (with 95% CI: 22.9, 32). In addition, a notable number (41.7%) of the respondents had ever heard about BSE. Nearly one-fourth (22.9%) of the study participants knew how to perform BSE, and about 43.5% respondents said that a girl should start BSE at the age of between 20 and 30. The BSE methods as reported by students include inspection (3.6%), palpation (17.4%), and both inspection and palpation (28.6%) (Table 2).

Concerning BSE practice, the overall proportion of students having good practice on BSE was found to be 17.4% (with 95% CI: 13.8, 21.6), and about 20.6% of the participants reported that they had ever practiced BSE (Table 3).

### 3.3. Factors Associated with Outcome Variables

All explanatory variables had been tested in the bivariable logistic regression with each outcome variable (i.e., knowledge and practice on BSE). Those variables with a *p* value of 0.2 had been then fitted to the multivariable logistic regression model.

According to the multivariable analysis result, the independent predictors of good knowledge on BSE were urban residence (AOR = 2.50; 95% CI: 1.27, 4.94) and ever discussed with someone on BSE (AOR = 4.50; 95% CI: 2.42, 8.65) (Table 4). On the other hand, determinants of good practice on BSE were identified to be family history of BC (AOR = 7.14; 95% CI: 1.75, 25), discussion with someone on BSE (AOR = 3.85; 95% CI: 1.82, 8.33), and good knowledge on BSE (AOR = 12.02; 95% CI: 5.97, 24.20) (Table 5).

## 4. Discussion

Breast cancer screening is one potentially important strategy in achieving early detection of cancer thereby facilitating early treatment and reducing women's death of BC [12]. Most healthcare facilities in Ethiopia do not have advanced laboratory investigations for screening and diagnosing of BC. Fortunately, BSE is an alternative cost effective and feasible screening strategy which is easily workable in any place and time in resource-limited settings [5]. Hence, this study assessed knowledge and practice on BSE and associated factors among female summer class university students.

In this study, the proportion of students with good knowledge on BSE was 27.6%. This finding is comparable with studies done in Debre Berhan, Ethiopia (30%) [16], Eretria (30.1%) [18], and Nepal (27%) [19]. However, the proportion of knowledge in this study is much lower than what was reported in India (93.3%) [17], Korea (87.0%) [20], Saudi (79.0%) [21], Uganda (76.5%) [22], Nigeria (55.4%) [23], and Egypt (63.4%) [24]. It is also lower than local reports in Ethiopia such as in Bahir Dar (75.3%) [11], Harare (93.6%) [9], and Jimma (89%) [25]. The observed discrepancy could be due to the difference in socioeconomic and demographic characteristics among the study population and difference in professions among the study participants. On the contrary, the magnitude of good knowledge on BSE is higher than that of reported in Adama University (8.7%) [5]. This disparity could be attributed to the high cut-off point (80-100%) in the operational definition of “good knowledge” in the former study [5] unlike that of ≥40% in the current study.

According to the current analysis findings, respondents' BSE knowledge is independently predicted by being urban resident and having ever discussion with someone else on BSE. The odds of having good knowledge on BSE were 2.5 times higher among urban residents than rural dwellers. This finding is supported by previous study done at Jimma University [25]. The possible explanation for this association could be due to the fact that respondents who reside in the urban area are likely to have better access to source of information such as mass media, Internet, and health care professionals. Similarly, good BSE knowledge is also predicted by ever discussion with someone on BSE. Hence, participants who ever discussed with someone on BSE were 4.5 times more likely to have good knowledge as compared to their congruent. This is possible as participants who discussed with someone on BSE are likely to gain more information than others.

In this study, about one in six (17.3%) of students had better BSE practice. The finding is similar with that reported in the study done in Silicon Valley of India (19.1%) [26], nationwide survey in Korea (16.1%) [20], and Addis Ababa university in Ethiopia (21.4%) [27]. The agreement in magnitude might be ascribed to the nonhealth profession of the respondents in common [26, 27].

On the other hand, the magnitude of BSE practice is lower than results reported from studies done in Indira Gandhi Medical College in India (54%) [17], systematic review in India (55.5%) [28], Abakalid in Nigeria (37.4%) [29], Hanoi and Chi Minh city in Vietnam (39.9%) [30], Hamedan in Iran (26%) [31], Uganda (43.6%) [22], and Poland (56.1%) [32]. It is also lower than local studies reported at different parts of Ethiopia such as Harar (23%) [9], Adama (39.4%) (2), Debre Birhan (28.3%) [16], Debre Tabor (32.5%) [33], West Shoa (32.6%) [34], Bahir Dar University (54.1%) [11], West Gojam Zone (37%) [13], and two studies in Addis Ababa (24.3% [35] and 21.4% [27]). The inconsistency in figure could be accredited to variation in study population that most of the participants in the previous studies are health professions who are likely to have better awareness towards BSE through their academic courses and trainings than nonhealth professionals [11, 13, 32–34].

On the contrary, the proportion of BSE practice is higher as compared to the reports of previous studies done in Egypt (7.4%) [24] and Eritrea (11.7%) [18]. The observed disagreement in magnitude might be attributed to dissimilarity in sociodemographic and cultural characteristics of the respondents.

The study revealed that BSE practice is significantly associated with family history of BC, discussion with someone on BSE, and knowledge on BSE.

The current study revealed that the odds of having good practice of BSE were 7.14 times higher among students with family history of BC than those who had no. This finding is also supported by previous studies [14, 15, 29]. This could be explained by the fact that students having family history of BC are more likely to get the opportunity to notice the burden as well as the preventive and screening measures of BC including BSE than their counters.

By logic, having free discussion with someone on the important common public health issues is anticipated to foster awareness on that particular matter. The current analysis advocated the importance of carrying out free discussion on BSE. Accordingly, respondents who ever discussed with someone on BSE were 3.85 times more likely to do better BSE practice as compared to those students who had never discussed. Similar finding was also reported from the studies conducted in West Gojam Zone [13]. This could be explained by the reality that participants who discussed with someone on BSE are likely to gain more information than others. This statistical association suggests that conducting a public discussion forum on BSE would escalate practice of BSE among women.

Evidences support that people with better knowledge towards health promotion actions are more likely to have better compliance with endorsed disease-preventive practices such as BSE [30, 31, 36]. The result of current analysis also corroborates this fact. Thus, students having good knowledge on BSE were 12.02 times more likely to perform better BSE practice compared with those with poor knowledge. This finding is in agreement with results reported from studies conducted in India [26] and Nigeria [29]. It is also aligned with the findings of local studies done in Addis Ababa [27], Debre Tabor [33], Debre Birhan University [16], and West Shoa [34] and SR and MA in Ethiopia [14, 15]. This might be due to the logic that students with better knowledge on the way how to do, the time when to do, and the purpose why to do BSE are more likely to test their health status by themselves in this particular issue and are likely to be enthused to know the result. This implies that awareness creation towards BSE is a pivotal part of prevention, early diagnosis, and treatment of breast cancer.

Finally, the authors would like to recommend that readers need to interpret the findings with cautions as the study recruited more specific study participants.

## 5. Conclusion

This study revealed that the proportion of good knowledge and practice of BSE among students was lower than many of other previous studies. The odds of having good knowledge were higher among respondents who resided in urban areas and ever had discussion on BSE. On the other hand, the independent predictors of good BSE practice included family history of BC, ever discussion with someone on BSE, and good knowledge on BSE. Thus, stakeholders need to work more on awareness creation on BSE taking the identified predictors of good BSE knowledge and practice into account. Moreover, the University of Gondar would better rearrange and deliver a planned tutorial on BSE targeting all female students. The authors also recommended further studies with qualitative approach to be carried out so as to explore the deep rooted barriers and facilitators of BSE practice.

## Figures and Tables

**Table 1:** Sociodemographic and general characteristics of summer social science female students in University of Gondar, Northwest Ethiopia, 2018 (*n* = 384).

Variables	Number	Percent
Age		
20-25	117	30.5
26-31	167	43.5
≥32	100	26.0
Residence		
Urban	218	56.8
Rural	166	43.2
Marital status		
Single	161	41.9
Ever married	223	58.1
Education level		
1^st^ & 2^nd^ year degree	105	27.3
3rd year degree and above	279	72.7
Payment status		
Free	179	46.6
Payee	205	53.4
Occupation		
Government employee	303	78.9
Student	81	21.1
Personal history of breast cancer		
Yes	25	6.96
No	359	93.4
Family history of breast cancer		
Yes	20	5.2
No	364	94.8
History of antenatal care visits		
Yes	92	24.0
No	292	76.0
History of contraceptive use		
Yes	185	48.2
No	199	51.8
Radio/television at home		
Yes	299	77.9
No	85	22.1
Ever discussed with someone on		
BSE	64	16.7
Yes	320	83.3
No		
Any breast complication		
Yes	26	6.8
No	358	93.2

**Table 2:** Knowledge on breast self-examination among summer social science female students in University of Gondar, Northwest Ethiopia (*n* = 384).

Variable	Number	Percent (%)
Heard about breast self-examination		
Yes	160	41.7
No	224	58.3
Know how to perform BSE		
Yes	88	22.9
No	296	77.1
Know appropriate time to perform BSE		
Yes	47	12.2
No	337	87.8
What do we look for during BSE		
Breast lamp	64	16.7
Size of the breast	42	10.9
Change in nipple and unusual discharge	22	5.7
All	101	26.3
I do not know	155	40.4
When should a girl begin		
At age less than 20 years	74	19.3
At age 20 to 30 years	167	43.5
At age above 30 years	87	22.7
I do not know	56	14.6
Which examination technique applied during BSE		
Inspection	14	3.6
Palpation	67	17.4
Both inspection and palpation	110	28.6
I do not know	193	50.3
Finding and outcome of doing breast self-examination		
Detect any abnormality	19	4.9
Learn how the breast normally looks and feels	21	5.5
Detect breast cancer earlier and promote treatment	51	13.3
All	267	69.5
I do not know	26	6.8

**Table 3:** Practice of breast self-examination among summer social science female students in University of Gondar, Northwest Ethiopia, 2018 (*n* = 384).

Variable	Number	Percent
Ever practice BSE		
Yes	79	20.6
No	305	79.4
Able to perform BSE confidently		
Yes	64	16.7
No	320	83.3
Perform BSE without clothes on		
Yes	53	13.8
No	331	86.2
Perform BSE in front of the mirror		
Yes	26	6.8
No	358	93.2
Use correct part of fingers during BSE		
Yes	63	16.4
No	321	83.6
Can able to detect any abnormality		
Yes	111	28.9
No	273	71.1
Do BSE while bathing		
Yes	38	9.9
No	346	90.1
Raise one hand above the head		
Yes	52	13.5
No	332	86.5
Touch entire part of breast during BSE		
Yes	34	8.9
No	350	91.1
Perform BSE press firmly		
Yes	39	10.2
No	345	89.8

**Table 4:** Factors associated with knowledge of BSE among summer social science female students in University of Gondar, Northwest Ethiopia (*n* = 384).

Variables	*Knowledge of BSE*		COR (95% CI)	AOR (95% CI)
	Yes	No		
Age				
20-25	14	103	0.54 (0.26, 1.14)	0.70 (0.27, 1.80)
26-31	31	136	0.91 (0.49, 1.71)	0.79 (0.38, 1.66)
≥32	20	80	1	1
Residence				
Urban	76	142	2.426 (1.496, 3.935)	2.50 (1.27, 4.94)^∗^
Rural	30	136	1	1
Education level				
1^st^ & 2^nd^ year degree	14	91	0.69 (0.36, 1.30)	0.88 (0.42, 1.85)
3^rd^ year degree and above	51	228	1	1
Occupation				
Government employee	55	248	1.57 (0.76, 3.25)	1.95 (0.80, 4.77)
Student	10	71	1	1
Payment				
Free	37	142	1.65 (0.96, 2.82)	1.05 (0.55, 2.01)
Payee	28	177	1	1
Marital status				
Single	23	138	0.72 (0.41, 1.25)	1.27 (0.57, 2.87)
Ever married	42	181	1	1
Family history of breast cancer				
Yes	8	12	1.81 (0.718, 4.559)	1.43 (0.38, 5.38)
No	98	266	1	1
Prenatal history				
Yes	35	71	0.523 (0.318, 0.801)	0.74 (0.34, 1.58)
No	57	221	1	1
Radio TV and other media at home				
Yes	91	208	2.04 (1.11, 3.75)	0.60 (0.23, 1.55)
No	15	70	1	1
Contraceptive history				
Yes	52	133	1.05 (0.67,1.643)	1.44 (0.67, 2.98)
No	54	145	1	1
Discussed with someone on BSE				
Yes	37	27	4.98 (2.84, 8.75)	4.57 (2.42, 8.65)^∗^
No	69	251	1	1

**Table 5:** Factors associated with practice of BSE among summer social science female students in University of Gondar, Northwest Ethiopia, 2018 (*n* = 384).

Variables	*BSE practice*		COR (95% CI)	AOR (95% CI)
	Good	Poor		
*Age (in year)*				
20-25	8	109	0.74 (0.27, 2.00)	2.15 (0.53, 8.78)
26-31	19	148	1.29 (0.56, 2.99)	1.53 (0.52, 4.53)
≥32	9	91	1	1
*Residence*				
Urban	22	196	1.22 (0.60, 2.46)	1.084 (0.53, 2.21)
Rural	14	152	1	1
*Education level*				
1^st^&2^nd^year degree	43	174	1.47 (0.85, 2.540)	1.98 (0.97, 4.04)
3rd year degree and above	24	143	1	1
*Occupation*				
Government employee	29	274	0.92 (0.38, 2.12)	1.708 (0.60, 4.79)
Students	7	74	1	1
*Payment*				
Free	39	140	01.76 (1.03, 3.00)	1.223 (0.60, 2.48)
Payee	28	177	1	1
*Marital status*				
Single	20	141	0.531 (0.301, 0.938)	0.85 (0.36, 2.06)
Ever married	47	176	1	1
*Family history of breast cancer*				
Yes	8	12	8.00 (3.02, 21.19)	7.14 (1.75, 25)^∗^
No	28	336	1	1
History of antenatal care visit				
Yes	12	80	1.67 (0.80, 3.50)	0.71 (0.32, 1.57)
No	24	268	1	1
Radio/television at home				
Yes	33	266	3.39 (1.02, 11.34)	0.74 (0.27, 2.02)
No	3	82	1	1
Contraceptive history				
Yes	17	168	0.96 (0.48, 1.91)	0.90 (0.40, 2.00)
No	19	180	1	1
Ever discussed with someone on				
BSE	23	41	13.25 (6.23, 28.16)	3.85 (1.82, 8.33)^∗^
Yes	13	307	1	1
No				
Knowledge status on BSE				
Good	23	42	12.89 (6.07, 27.36)	12.02 (5.97, 24.20)^∗^
Poor	13	306	1	1

1 = reference; ^∗^*p* value < 0.05.

## Data Availability

The datasets employed in the current study can be available from the corresponding author upon the reasonable request.

## Abbreviations

BC: Breast cancer
BSE: Breast self-examination
SR: Systematic review
MA: Meta-analysis.
